# Development and Validation of a Culturally Adapted Patient‐Reported Experience Measure for Diabetes Care in Thailand: Mixed‐Methods Study

**DOI:** 10.1111/hex.70619

**Published:** 2026-02-27

**Authors:** Soe Sandi Tint, Nida Buawangpong, Wichuda Jiraporncharoen, Myo Zin Oo, Nutchar Wiwatkunupakarn, Kittipan Rerkasem, Kanokwan Kulprachakarn, Hataichanok Chuljerm, Timothy E. O'Brien, Rohini Mathur, Petch Rawdaree, Chaisiri Angkurawaranon

**Affiliations:** ^1^ Global Health and Chronic Conditions Research Center Chiang Mai University Chiang Mai Thailand; ^2^ Department of Family Medicine, Faculty of Medicine Chiang Mai University Chiang Mai Thailand; ^3^ School of Health Sciences Research, Research Institute for Health Sciences Chiang Mai University Chiang Mai Thailand; ^4^ Substance Use Research Unit, Research Institute for Health Sciences Chiang Mai University Chiang Mai Thailand; ^5^ Environmental‐Occupational Health Sciences and Non‐Communicable Diseases Research Center, Research Institute for Health Sciences Chiang Mai University Chiang Mai Thailand; ^6^ Clinical Surgical Research Center, Department of Surgery, Faculty of Medicine Chiang Mai University Chiang Mai Thailand; ^7^ Department of Mathematics and Statistics Loyola University Chicago Chicago Illinois USA; ^8^ Wolfson Institute for Population Health University of London London UK; ^9^ Faculty of Medicine, Vajira Hospital Navamindradhiraj University Bangkok Thailand; ^10^ Diabetes Association of Thailand Bangkok Thailand

**Keywords:** diabetes care, diabetes mellitus, patient reported experience measures, patient‐centred care

## Abstract

**Background:**

Patient‐centred care is essential for managing Type 2 diabetes, as it relies on understanding patients’ experiences and perspectives. However, in Thailand, no culturally adapted patient‐reported experience measure (PREM) exists to capture these insights and support quality improvement in diabetes care.

**Objective:**

To develop and validate a culturally adapted PREM for Thai people with Type 2 diabetes receiving primary care.

**Methods:**

This mixed‐methods study was conducted at Saraphi Hospital, Chiang Mai, from July to December 2024. Stage 1 involved drafting the initial PREM based on a prior scoping review. In Stage 2, content validity was assessed by experts, and face validity was evaluated through patient interviews. Stage 3 involved a quantitative survey of patients to assess construct validity and reliability using exploratory and confirmatory factor analyses.

**Results:**

The initial PREM included 37 items across four domains, developed based on findings from the scoping review. After expert and patient feedback, the tool was refined. Exploratory and confirmatory factor analyses confirmed the tool's structure, resulting in a final version with 16 items across four domains: care planning, patient education, professionalism and quality of service. This final PREM structure explained 60.1% of overall variance, and demonstrated good internal consistency (Cronbach's alpha of 0.84), supporting its utility for capturing patient experiences in the Thai healthcare setting.

**Conclusion:**

This study developed the first culturally adapted PREM for Thai people living with diabetes. Although further implementation is needed, this current validated tool can support patient‐centred care in Thailand and other low‐ and middle‐income settings in Southeast Asia.

**Patient or Public Contribution:**

The development of this instrument actively involved patients throughout key stages. During content validation, individuals living with diabetes participated in an expert panel and provided feedback on item clarity, relevance and comprehensiveness. Their suggestions directly influenced revisions to the questionnaire. For face validity, a pilot test was conducted with patients in a primary care setting, gathering their insights on wording, usability and cultural appropriateness. Their contributions ensured the tool was clear, acceptable and contextually relevant to Thai people living with diabetes.

## 1 Introduction

Patient‐reported experience measures (PREMs) are essential tools for evaluating the quality of healthcare services from the patient's perspective [1]. Unlike patient‐reported outcome measures (PROMs), which focus on clinical outcomes such as symptoms and functional status [1], PREMs assess experiences with care delivery, including communication, shared decision‐making, and accessibility of services. In chronic disease management, particularly for diabetes mellitus (DM), PREMs are particularly valuable for capturing patient engagement, self‐management support, and the overall effectiveness of healthcare services [2]. These experiences are typically measured using validated self‐reported tools, such as standardized questionnaires or surveys, allowing patients to evaluate their experiences with healthcare without third‐party interpretation directly [3].

Several PREMs have been developed to assess patient experiences across various healthcare settings, including both general and disease‐specific PREMs. General PREMs, such as the Consumer Assessment of Healthcare Providers and Systems (CAHPS) [4] and the Patient Experience Questionnaire (PEQ) [5], evaluate overall healthcare experiences across different patient populations [6]. In contrast, disease‐specific PREMs focus on particular conditions, capturing experiences relevant to disease management and treatment. For example, the Patient Assessment of Chronic Illness Care (PACIC) [7] is commonly used for chronic disease management, including diabetes care. In Thailand, a general PREM was launched in December 2024 by the Institute for Accreditation of Healthcare Facilities [8]; however, its application remains limited. Furthermore, there is a clear lack of disease‐specific PREMs, particularly for individuals living with diabetes. Since diabetes care involves ongoing patient–provider interactions, effective self‐management and coordination across various healthcare services [9], a diabetes‐specific PREM is essential to capture patient experiences and identify areas for improvement accurately.

Several PREMs have been developed in different countries to assess patient experiences with diabetes care, including the UK, Sweden, France, Norway and Denmark, with only one study from a middle‐income country, Ecuador [10]. However, many of these instruments have undergone limited psychometric testing, with a particular gap in dimension‐level reliability assessments, raising concerns about whether these tools consistently measure specific aspects of patient experience [10]. Without a robust psychometric evaluation, including tests of internal consistency and reliability at the domain level, the applicability of these instruments across different cultural and healthcare settings remains uncertain [11]. This limitation is particularly significant when considering adaptation to the Thai context, where patient expectations, communication styles, and healthcare delivery models differ substantially from those in high‐income countries. Therefore, developing a culturally tailored PREM that undergoes rigorous psychometric validation is essential to ensure an accurate and meaningful assessment of patient experiences in Thailand.

Primary healthcare is delivered in Thailand through a tiered system comprising sub‐district health promotion hospitals (SHPHs), district hospitals, and health centres. SHPHs serve as the first point of contact for most patients and play a pivotal role in delivering accessible diabetes care close to patients’ communities [12]. To improve access and reduce congestion at larger hospitals, the Ministry of Public Health has implemented a national primary care policy expanding diabetes care services at SHPHs [13]. However, despite these efforts, optimal outcomes remain difficult to achieve due to some barriers, including limited communication between patients and providers, often focused narrowly on clinical indicators like blood glucose levels, while patients’ concerns and daily challenges are overlooked. Additionally, many patients receive insufficient education on how to manage their condition independently, which hampers effective diabetes self‐care in primary care settings [14]. Additionally, healthcare providers in primary care settings face high patient loads and resource constraints while navigating diverse patient needs within complex social and cultural contexts [15].

Given these challenges, there is a pressing need for culturally adapted, disease‐specific PREMs tailored to DM patients in Thai primary care settings. Such tools could provide insights into patient experiences with long‐term diabetes management while addressing gaps in communication, education and resource allocation. A well‐validated PREM could not only enhance understanding of patient experiences but also support improvements in patient‐centred care practices across Thailand. Therefore, this study aims to address the gap by developing a culturally adapted, disease‐specific PREM for people living with diabetes, designed for use in primary care settings across Thailand.

## 2 Methods

The development of the PREM for people living with diabetes was carried out in three stages, utilizing both quantitative and qualitative methods, at Saraphi Hospital, Chiang Mai, Thailand, from July to December 2024. Saraphi Hospital is a district‐level hospital that provides primary care services, including noncommunicable disease (NCD) clinics, infectious disease clinics and antenatal care (ANC) clinics. This study was conducted at the NCD clinic, which holds outpatient sessions twice a week for people living with diabetes. The clinic serves an average of 1500 to 2000 people living with diabetes, offering primary care services to manage their condition. Although this study was limited to a single site, the demographic and clinical characteristics of the sample, such as age distribution, gender and duration of diabetes, are broadly reflective of the Thai type 2 diabetes population receiving care at the primary level. This is because, under Thailand's Universal Health Coverage (UHC) scheme, most individuals with chronic conditions, like diabetes, receive regular care through primary care units and district hospitals near their residence [16]. These facilities play a key role in managing non‐communicable diseases [13], making the patient population at Saraphi Hospital broadly comparable to those in similar settings across the country.

**Figure 1:**
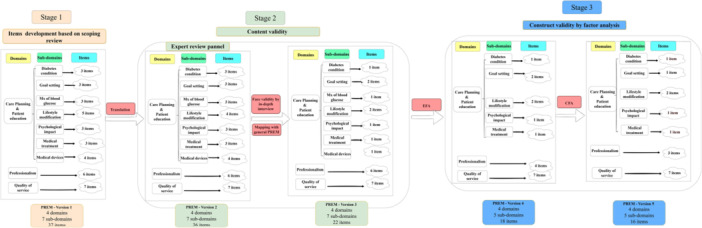
Overview of the development process of PREM‐DM‐Thai. *Domain: Broad categories that represent key areas of patient experience with healthcare services. *Subdomain: More specific aspects within each domain that capture distinct elements of patient experience. *Item: Individual questions or statements designed to assess patient experience related to each subdomain or domain.

Figure 1 provides a detailed description of each component and an overview of the development stages and processes. It also highlights the number of items in each stage and the differences between each version of the tool.

### 2.1 Stage 1: Item Development

#### 2.1.1 Initial Questionnaire Development

The PREM questionnaire was developed based on findings from a scoping review previously conducted by our team [10], which highlighted key areas of patient experience that should be measured. These areas, called domains, include care planning, patient education, professionalism, and quality of service [10]. Specifically, ‘a domain or construct refers to the concept, attribute, or unobserved behaviour that is the target of the study’ [17]. Each domain was broken down into more specific topics, known as subdomains. For example, within care planning, one subdomain is goal setting. To measure patient experience within each area, we created items—these are individual questions or statements that patients respond to [17]. For example, under the sub‐domain ‘goal setting’, we included items such as: ‘Did you agree on your care planning with your healthcare provider regarding the goal setting of your diabetes care?’ These are referred to as items. Initially, we gathered over 100 items from existing questionnaires. Most items were revised to improve clarity, enhance cultural relevance, and fit the Thai healthcare context. The initial adaptation was conducted collaboratively by the principal investigator, one NCD researcher, two healthcare professionals experienced in diabetes care from this research group, and one bilingual translator from the research team. The cultural adaptation process involved simplifying technical terms into everyday Thai language, rephrasing questions to reflect typical communication styles between Thai patients and healthcare providers, and adding locally relevant examples to facilitate patient understanding. After removing overlaps and refining the wording, we finalized a version of the questionnaire that includes 4 domains, 7 subdomains and 37 items in total. Each item is rated using a 5‐point Likert scale, from ‘(1) not at all’ to ‘(5) to a very large extent.’ This simple scale was chosen because it is easy for patients to understand and use in a primary care setting [18].

#### 2.1.2 Translation Process

The first version of the PREM was initially discussed within the research group to evaluate its relevance, wording, and structure. The questionnaire's layout and content were revised based on the group's recommendations. Since the first version was developed in English, the following steps were undertaken to translate and culturally adapt the PREM for Thai people living with diabetes. **Forward translation**: The revised English version of the PREM was translated into Thai by a member of the research team who is a native Thai speaker, has a degree in English, and has over 5 years of experience in medical translation. This step ensured linguistic accuracy and contextual relevance to Thai culture. **Backward translation**: The Thai version was sent to an independent translator, who had no prior exposure to the questionnaire, for translation back into English. This ensured that the meaning of the original questionnaire was preserved. **Reconciliation**: The research team compared the backward translation with the original English version to identify discrepancies. Any differences were discussed and resolved, ensuring conceptual equivalence between the English and Thai versions. **Revision of the Thai version** *:* After completing the forward and backward translations, the Thai version was reviewed again to refine wording and structure, making it clearer and more culturally appropriate for Thai people living with diabetes.

### 2.2 Stage 2: Content and Face Validity

#### 2.2.1 Content Validity by Expert Panel

The first version of the Thai PREM was evaluated by a panel of 13 Thai experts, including physicians, nurses, researchers specializing in NCDs, NCD clinic case managers, statisticians and people living with diabetes. The experts assessed the questionnaire across three dimensions—clarity, relevance, and comprehensiveness—using a 4‐point scale, where a score of 4 indicated high relevance and a score of 1 indicated low relevance, and for comprehensiveness [19], experts provided additional comments and suggestions for each item.

Following the evaluation, the item‐level content validity index (I‐CVI) and scale‐level content validity index (S‐CVI) were calculated. Based on established benchmarks, an I‐CVI value of ≥0.78 and an S‐CVI value of ≥0.90 were considered acceptable [19]. In this study, all items met the recommended thresholds. However, based on expert feedback, two items under the ‘Lifestyle Modification’ subdomain were combined into a single item. Initially, these items separately addressed discussions with healthcare providers regarding dietary changes and physical activity. They were merged into a broader question: ‘To what extent did you discuss lifestyle modifications with your healthcare provider, including diet, physical activity, foot care, and medication adherence?’ Additionally, minor wording refinements and the inclusion of examples were made to enhance clarity. Following expert validation, the second version of the PREM consisted of 4 domains, 7 subdomains, and 36 items.

#### 2.2.2 Face Validity

The second version of the PREM DM Thai was pilot‐tested among 20 people living with Type 2 diabetes. Purposive sampling was used to select patients from the Saraphi district hospital who had received care at the diabetes clinic for at least 1 year. On clinic days, the data collection team approached patients, verified their eligibility by checking their records, and explained the study to those who met the criteria. Informed consent was obtained from those who agreed to participate, and individual in‐depth interviews were conducted by research assistants from the data collection team. During the interviews, participants first completed the PREM DM Thai questionnaire independently. They were then asked about their understanding of the wording, the ease or difficulty of the questions, their ability to answer without assistance, and any suggestions for improving the wording. Overall, participants found the tool clear and appropriate, and no modifications were made following the pilot test.

#### 2.2.3 Mapping With the General PREM of Thai

The second version of the PREM, consisting of 36 items, was compared with the general PREM used in the national patient experience program organized by the Health Accreditation Organization of Thailand, which consists of 17 items [8], to identify differences and determine which items should be retained or removed. This process aimed to ensure the development of a disease‐specific PREM tailored to the experiences of patients with diabetes.

Following this comparison, items not included in the general PREM but found to be relevant for diabetes care were retained or modified, while those that patients found difficult to understand during interviews were removed. As a result, the third version of the PREM‐DM Thai was finalized, consisting of four domains, seven subdomains, and 22 items.

### 2.3 Stage 3: Construct Validity

#### 2.3.1 Construct Validity by Factor Analysis

In the final stage of validation, construct validity was assessed through the use of factor analysis [20]. The third version of the PREM‐DM Thai, consisting of 22 items, was administered to 220 people living with Type 2 diabetes at Saraphi Hospital from August to October 2024. Participants were recruited using convenience sampling; patients attending the outpatient diabetes clinic during the study period were approached by the research team. Only those who had received care for at least 1 year, were able to communicate in Thai, and provided informed consent were included. Patients with significant cognitive impairment, severe psychiatric illness or acute medical instability were excluded. Among the eligible patients approached, some declined participation; however, the number of refusals (non‐uptake) was not systematically recorded. Among those who agreed to participate, all completed the questionnaire in full, resulting in no incomplete responses. Research assistants from our team were available to clarify any questions and ensure that every item was answered, supporting the completeness and quality of the data. ‘Thai’ identity was defined by self‐identification and the ability to fluently communicate in Thai. All participants in this study were identified as of Thai ethnicity. The sample size was determined based on established guidelines recommending at least 10 participants per item [17]. Data analysis was performed using Stata version 15.

Exploratory factor analysis (EFA) was first conducted on the third draft of the PREM‐DM Thai, which consisted of 22 items, to identify its underlying factor structure. Factors were extracted using principal axis factoring, with oblique rotation applied to account for potential correlations between factors. The number of factors retained was determined based on the proportion of variance explained, parallel analysis results, and clinical relevance. Items were considered to load onto a factor if the pattern matrix correlation was ≥0.4, with cross‐loading identified when item loadings differed by ≤0.2 across factors [21].

The factor structure identified in the EFA was then confirmed through confirmatory factor analysis (CFA). Model fit was assessed using multiple indices, including the chi‐square statistics, the comparative fit index (CFI) and the root mean square error of approximation (RMSEA). A good model fit was indicated by RMSEA < 0.06 and CFI ≥ 0.95, while an acceptable fit was defined as RMSEA < 0.08 and CFI ≥ 0.90 [22]. The internal consistency was evaluated using composite reliability (CR), with values ≥0.7 indicating good reliability [23]. To determine whether a total score for the PREM‐DM‐Thai was appropriate, the correlations between factors were examined, and a second‐order model was tested. The nested Chi‐square difference test and model fit indices were used to compare the first‐order and second‐order models.

### 2.4 Reliability and Validity

Internal consistency of the PREM‐DM‐Thai subscales was assessed using Cronbach's alpha. A value of ≥0.70 was considered acceptable, while values below this threshold were interpreted cautiously, given the small number of items in some subscales [24].

The relationships between subscales were examined using Pearson's correlation coefficients. Correlation values were interpreted as follows: low (0.10–0.29), moderate (0.30–0.49), and high (≥0.50). A significance level of *p* < 0.001 was applied to account for multiple comparisons [25].

## 3 Results

### 3.1 Construct Validity

The third version of the PREM‐DM Thai, consisting of 22 items, was tested among 220 patients using statistical methods to finalize the questionnaire. In this step, most of the study participants were female, 61.4%, and the average age of participants was between 56 and 74 years old. According to the responses from people living with diabetes in this study, 80.9% have hypertension, and 62.3% have hyperlipidemia. Table 1 describes the characteristics of patients who participated in this step.

**Table 1:** Characteristics of participants (*N* = 220).

Characteristics	Number (*N* = 220)	%
Gender		
Male	85	38.6
Female	135	61.4
Age		
Mean age ± SD (min–max)	65.1 ± 8.79	(34–84)
Marital status		
Single, never‐married	23	10.5
Married	133	60.4
Widowed	49	22.3
Divorced	15	6.8
Education		
No formal education	8	3.6
Primary education	144	65.5
Secondary education	51	23.2
Higher education	17	7.7
Occupation		
Government staff	4	1.8
Own business/Private company staff	51	23.2
Dependent/Unemployed	61	27.7
Household service worker	37	16.8
Others	67	30.5
Other chronic conditions		
Hypertension	178	80.9
Hyperlipidemia	137	62.3
Others	38	17.3

#### 3.1.1 Exploratory Factor Analysis

The initial EFA included 22 items, conducted using principal component factor analysis with varimax rotation, which helps simplify the factor structure by maximizing the variance of factor loadings [26]. A factor loading threshold of 0.40 was set, meaning that only items with a loading of 0.40 or higher were considered significant in representing the underlying factors [27]. This threshold ensures that only items with a moderate to strong relationship with the factor are retained, helping to create a clearer and more reliable factor structure.

In the first iteration, question number 5 under the professionalism domain exhibited cross‐loadings and was conceptually ambiguous in representing a single factor, leading to its removal. Also, question number 2 under the medical device domain had factor loadings near the 0.40 threshold and was considered redundant based on content overlap with another item, so it was also excluded. These two items were removed, reducing the item set to 20 items. Details of item loadings, decisions on item retention or deletion, and full EFA results for each step are presented in Supporting Information File 1.

A second EFA iteration led to removing question number 6 under the professionalism domain to maintain consistency with the predefined domain structure. In addition, question number 2 under the patient education domain, within the sub‐domain of management of blood glucose, was also deleted due to all its factor loadings being below the 0.40 threshold. This step, along with the previous item reductions, is also detailed in Supporting Information File 1. The final EFA solution retained 18 items, and the scree plot confirmed the retention of four factors. The four retained factors explained a total of 60.10% of the variance, with Factor 1, 24.65%, Factor 2, 14.31%, Factor 3, 13.67% and Factor 4, 7.47% of the total variance. The rotated factor loadings (pattern matrix) are presented in Table 2, showing how each variable is loaded onto the four factors with 18 items.

**Table 2:** Rotated factor loadings (pattern matrix) for the final 18 items from exploratory factor analysis.

Variable	Factor 1	Factor 2	Factor 3	Factor 4
Did you agree on your care planning with your health care provider about the goal setting of your diabetes care? **goal_q2**	0.0442	**0.5482**	−0.2274	0.1239
Were you offered a written, printed or electronic copy of your care plan? **goal_q3**	−0.0211	−0.1491	−0.0311	**0.7874**
Did you agree on your care planning with your health care provider about a plan for lifestyle modification? **life_q3**	0.1602	**0.8033**	0.2452	−0.0712
Did you receive useful and enough information from the health care provider about your disease condition? **dis_condit2**	0.1611	**0.5136**	−0.092	0.5095
Did you receive useful and enough useful information from the health care provider about the lifestyle modification? **life_q2**	0.1145	**0.8165**	0.2879	−0.0746
Did you receive useful and enough information from the health care provider about the psychological impact of diabetes on your daily life? **psy_q2**	0.0231	0.1173	0.365	**0.5846**
Did you receive useful and enough information from health care provider about the diabetes medication and treatment? **tx_q2**	**0.4278**	**0.5759**	−0.044	0.1409
To what extent did your healthcare provider explain diabetes‐related information to you clearly? **pro_q1**	**0.4402**	0.2226	**0.4118**	−0.0326
To what extent did your healthcare provider listen carefully to your concerns and questions? **pro_q2**	0.1387	0.1314	**0.8751**	0.0099
To what extent did your healthcare provider effectively address your concerns and questions about diabetes management? **pro_q3**	0.182	0.127	**0.8421**	0.0209
To what extent did you feel respected and treated with dignity during your interactions with healthcare providers? **pro_q4**	**0.4994**	0.1896	**0.4745**	0.1302
How satisfied are you with the accessibility of the care structure? (e.g., transportation, parking, etc.) **qos_q1**	**0.7326**	0.1643	0.0215	0.0464
How satisfied are you with the clinic environment (e.g., cleanliness, comfort, and overall atmosphere)? **qos_q2**	**0.7151**	0.0717	0.2138	−0.0354
How satisfied are you with the communication with the healthcare provider during the consultation? **qos_q3**	**0.7428**	0.1587	0.3011	−0.1197
How satisfied are you with the overall waiting experience, including wait times before seeing the doctor, during consultation and after seeing the doctor (to get medicine, to pay at the cashier)? **qos_q4**	**0.7291**	−0.059	−0.0185	0.1919
How satisfied are you with the communication with the other clinic staff during your visit? **qos_q5**	**0.7515**	0.1609	0.2844	0.0245
How satisfied are you with the accessibility of your health care provider when you need help with diabetes care? (e.g., contact number, appointment when needed) **qos_q6**	**0.5912**	0.308	0.1594	−0.0553
How satisfied are you with the range of diabetes care services provided, such as blood tests, eye exams and foot checks? **qos_q7**	**0.8033**	0.1772	0.09	0.0122

*Note:* Bold values indicate factor loadings >= 0.40 and represent the primary factor for each item.

#### 3.1.2 Confirmatory Factor Analysis

Following EFA, CFA was conducted to validate the factor structure using structural equation modelling (SEM) [28]. The initial CFA model included 18 items, categorized into four latent constructs. Model fit indices were assessed using goodness‐of‐fit statistics, and modification indices were examined. The results indicated that question number 3 under goal setting of diabetes care did not contribute significantly to the model, leading to its removal. A revised CFA model with 17 items was initially tested, resulting in a CFI of 0.877 and an RMSEA of 0.087, both indicating suboptimal model fit (commonly accepted thresholds: CFI ≥ 0.90, RMSEA < 0.08). Modification indices suggested a correlation between two questions under the professionalism domain. This correlation was theoretically justified, as both items reflect the broader concept of professionalism in provider–patient interactions. To address the cross‐loading concerns and enhance model fit, one question was removed, and the CFA model was refitted with the suggested error correlation included. The final CFA model included 16 items, demonstrated good model fit (CFI = 0.919 and RMSEA = 0.070), and was distributed across the four PREM domains as described in Table 3. A detailed summary of how each item was deleted, revised or retained from the initial 37‐item version is provided in Supporting Information File 2.

**Table 3:** Final PREM‐DM‐Thai.

Items no.	**Domain 1: Care planning**
	Did you agree on your care planning with health care provider about the following:
1	Goal setting for your diabetes care
2	A plan for lifestyle modification
	**Domain 2: Patient Education**
	Did you receive useful and enough information from health care provider about the following:
3	Disease conditions
4	Lifestyle modification
5	The psychological impact of diabetes on your daily life
6	Diabetes medication and treatment
	**Domain 3: Professionalism**
7	To what extent did your healthcare provider explain diabetes‐related information to you clearly?
8	To what extent did your healthcare provider listen carefully to your concerns and questions?
9	To what extent did you feel respected and treated with dignity during your interactions with healthcare providers?
	**Domain 4: Quality of service**
10	How satisfied are you with the accessibility of the care structure? (e.g., transportation, parking, etc.)
11	How satisfied are you with the clinic environment (e.g., cleanliness, comfort and overall atmosphere)
12	How satisfied are you with the communication with the healthcare provider during the consultation?
13	How satisfied are you with the overall waiting experience, including wait times before seeing the doctor, during consultation and after seeing the doctor (to get medicine, to pay at the cashier)?
14	How satisfied are you with the communication with the other clinic staff during your visit?
15	How satisfied are you with the accessibility of your health care provider when you need help with diabetes care? (e.g., contact number, appointment when needed)
16	How satisfied are you with the range of diabetes care services provided, such as blood tests, eye exams and foot checks?

*Note:* The full set of questionnaires containing these 16 items in both English and Thai is available in Supporting Information File 3.

### 3.2 Reliability and Validity

The internal consistency of the PREM‐DM‐Thai subscales, measured using Cronbach's alpha, ranged from 0.36 (care planning) to 0.85 (quality of service), with an overall reliability of 0.84. The quality‐of‐service subscale showed the highest internal consistency, while the Care Planning subscale had a lower alpha value, likely due to having only two items.

Intercorrelations among subscales were all statistically significant at the 0.001 level (Table 4). The strongest correlation was observed between quality of service and both patient education and professionalism (*r* = 0.46), while the weakest correlation was between care planning and professionalism (*r* = 0.23).

**Table 4:** The PREM‐DM‐Thai subscale correlation matrix.

Factor (16 items)	Care planning (2 items)	Patient education (4 items)	Professionalism (3 items)	Quality of service (7 items)	Cronbach's alpha
Care planning (2 items)	1.00				0.36
Patient education (4 items)	0.49^a^	1.00			0.53
Professionalism (3 items)	0.23^a^	0.33^a^	1.00		0.76
Quality of service (7 items)	0.27^a^	0.46^a^	0.46^a^	1.00	0.85
Overall					0.84

^a^ 0.001.

## 4 Discussion

This study aimed to develop a PREM tailored specifically for Thai patients with DM in primary care settings. The development process was built on findings from a previously published scoping review, followed by expert consultations and extensive validation to ensure the instrument's relevance, clarity and cultural appropriateness. Notably, patients were actively involved throughout the process, contributing their perspectives to enhance the instrument's relevance, clarity and cultural appropriateness—ensuring that it truly reflects patient experiences and priorities. Our findings contribute to the growing body of knowledge on patient‐reported experiences, particularly in the context of diabetes care, and highlight the need for culturally adapted, disease‐specific tools in Southeast Asia.

### 4.1 Addressing the Gap in Southeast Asia

According to scoping review results, PREMs have been developed in various healthcare settings worldwide to assess the quality of diabetes care [10]. However, a key limitation of many existing PREMs is that they do not comprehensively capture all critical domains of patient experience. Most existing PREMs focus on specific aspects of care rather than providing a holistic view of diabetes management from the patient's perspective. For example, the National Audit, Patient Experience of Diabetes Service Survey (PEDS) from the UK primarily assesses care planning and care provision [29], while the National Diabetes Register NDR in Sweden focuses mainly on support from healthcare providers and medical treatment [30]. These instruments, though valuable, may not fully encompass the breadth of patient experiences, particularly in areas such as self‐management support, shared decision‐making, health education, and accessibility of care. To address these limitations, the PREM developed in this study was designed based on a systematic scoping review of existing instruments, ensuring that it includes all key domains that are widely used in diabetes care experience assessments. By incorporating a broader range of domains—including care planning, patient education, provider communication, and service accessibility—this PREM offers a more comprehensive and culturally tailored tool for evaluating diabetes care in Thailand. This broader scope ensures that patient experiences are assessed more holistically, capturing not only interactions with healthcare providers but also the structural and personal factors influencing long‐term diabetes management. As a result, this PREM has the potential to provide more actionable insights for improving patient‐centred care in Thailand and could serve as a model for developing more comprehensive PREMs in other healthcare systems. Furthermore, unlike many existing instruments, this PREM has undergone a psychometric evaluation, including factor analysis and internal consistency testing, to ensure its reliability and validity. This makes it a potentially valuable tool for improving diabetes care in low‐ and middle‐income countries, especially within Southeast Asia.

Additionally, many existing instruments do not align with the cultural context of Southeast Asia, particularly in terms of patient–provider interactions, where cultural factors significantly influence patient behaviours. For example, the Adolescent Patient Experiences of Diabetes Care Questionnaire (APEQDC) study, from Norway, which explores shared decision‐making in diabetes care, assumes a more active patient role in discussing and influencing their care plans. However, this approach may not be entirely applicable in the Thai cultural context, where patients typically do not question doctors or actively engage in decision‐making regarding their treatment and management plans [31]. This cultural norm is influenced by factors such as social hierarchy, respect for authority figures and a general reluctance to engage in assertive communication with healthcare providers, regardless of the patient's education level [32].

This cultural characteristic was considered during the development of our Thai‐specific PREM, which does not emphasize shared decision‐making as strongly as other PREMs, such as those from the UK or Sweden [29, 30]. Instead, our instrument focuses on care planning, provider communication, trust and the patient's satisfaction with the information provided by healthcare professionals, aligning with the Thai cultural expectations that prioritize respectful listening and trust in the doctor's expertise. By reflecting the cultural dynamics of Thai society, this PREM offers a more accurate and relevant assessment of the patient experience in the context of Thai diabetes care.

While some instruments fail to align with Thai cultural norms, particularly in terms of patient‐provider interactions, other studies have highlighted similarities in key domains relevant to patient experiences with diabetes care. For example, the Ecuador study emphasizes the importance of information delivery, care delivery, and patient‐centred care—domains that are also central to our Thai PREM [33]. Like the instruments in ‘Diabetes‐specific patient reported experience and outcome measure (EDP questionnaire)’ from Ecuador, our tool captures patient experiences with the quality of information provided under the patient education domain, the efficiency of care delivery, and the level of patient‐centred care they receive, under the domains of professionalism and quality of services, ensuring that these critical factors are included for a comprehensive assessment of diabetes care.

Although this PREM reflects the cultural norms of Thai society and captures key domains of diabetes care experience, the study sample included only Thai patients from northern Thailand. Thailand is home to diverse ethnic and linguistic groups, including hill tribes such as the Karen, Hmong, Akha, Lahu, Lisu and Mien (Yao), who may have distinct healthcare experiences [34]. Moreover, the national diabetes population is heterogeneous, with regional variations in culture, language, and access to healthcare services. Future research should validate the instrument among these groups to ensure cultural and linguistic appropriateness and to strengthen its applicability across the broader Thai diabetes population.

### 4.2 Importance of Disease‐Specific PREMs

In Thailand, general PREMs have been introduced but remain underutilized, as discussed in the introduction section [8]. Moreover, patient satisfaction questions for diabetes management have been developed for both Thai and Lao PDR [35], but it is important to distinguish between patient satisfaction and patient experiences. Patient satisfaction is ‘about whether a patient's expectations about a health encounter were met’ [36], and patient experience ‘encompasses the range of interactions that patients have with the healthcare system, including their care from health plans, and from doctors, nurses, and staff in hospitals, physician practices, and other healthcare facilities’ [36]. Therefore, two individuals receiving the same care may provide different satisfaction ratings due to their varying expectations of how that care should be delivered. In the case of diabetes management, patient experience should include how patients feel about their ongoing care, how well they understand their treatment plans, and whether they feel empowered to manage their condition in the long term [10]. While general PREMs can provide some insight into the overall quality of care, they do not capture the nuances of managing a chronic condition such as diabetes, which requires continuous monitoring, patient education and complex treatment regimens [37]. The disease‐specific PREM developed in this study is therefore a significant advancement, offering a more precise measurement of patient experiences with diabetes care and informing improvements in service delivery.

### 4.3 Reliability and Validity of the PREM‐DM‐Thai

The development of the PREM‐DM‐Thai was supported by rigorous testing for reliability and validity. Our study found that the tool demonstrated good internal consistency, with Cronbach's alpha, with an overall reliability of 0.84, values exceeding acceptable thresholds across most subscales [11]. Despite a low Cronbach's alpha of 0.36, the Care Planning subscale was retained due to its critical role in capturing a fundamental aspect of patient‐centred diabetes care. This subscale encompasses essential elements such as goal setting and an individualized treatment plan. These components are integral to effective diabetes management and align with established frameworks in chronic disease care [38, 39]. Additionally, Cronbach's alpha can be artificially low for subscales with very few items, as alpha is influenced by the number of items [40]. Since the Care Planning subscale only contains two items, the low alpha does not necessarily reflect poor measurement quality but rather the subscale's brevity. Retaining this subscale ensures the instrument remains comprehensive and addresses all key dimensions of patient experience in diabetes care. These findings indicate that the PREM‐DM‐Thai is a reliable instrument for measuring the patient experience in diabetes care settings. Furthermore, the subscale correlations showed moderate to strong relationships between domains such as care planning, patient education, professionalism and quality of service, underscoring the interrelated nature of these dimensions in providing comprehensive care. These results indicate that different aspects of patient experience were moderately associated, supporting the conceptual structure of the instrument.

## 5 Limitations

While this study has made significant strides in developing a disease‐specific PREM for Thai people living with diabetes, several limitations should be considered. First, although this current study was conducted in district‐level primary care settings that are typical under Thailand's Universal Health Coverage (UHC) scheme, the limited number of study sites may not fully capture the diverse experiences of people living with diabetes across the country. All participants in this study identified as Thai ethnicity, and recruitment was limited to only one district hospital in northern Thailand. This may limit the generalizability of the findings to the wider Thai diabetes population, which includes diverse ethnic and linguistic groups as well as regional variations in healthcare contexts. Second, participants were recruited using convenience sampling, and the number of patients who declined participation was not systematically recorded, which may lead to potential selection bias. Finally, the EFA solution explained 60.1% of the total variance. While this is within accepted norms for health and social science questionnaire development [27], the modest proportion suggests that some variance in patient‐reported experiences may not be fully captured by the instrument.

## 6 Recommendations

Future studies should aim to validate the PREM‐DM‐Thai across a broader and more varied range of healthcare settings nationwide. In addition, longitudinal validation is recommended to assess the tool's stability over time and responsiveness to changes in care. Furthermore, as many countries in Southeast Asia share similar cultural norms, particularly regarding patient–provider interactions and health‐seeking behaviours, validating this tool in other Southeast Asian countries could enhance its applicability and usefulness at a regional level. This current PREM framework may also be adaptable to other chronic conditions, such as hypertension. Additionally, while we focused on the psychometric properties of the PREM, future research should also explore its feasibility in routine clinical practice and assess its impact on healthcare outcomes, such as patient satisfaction and quality of life.

## 7 Conclusion

The development of the PREM‐DM‐Thai is an important contribution to the field of diabetes care in Thailand. By capturing key aspects such as care planning, patient education, professionalism and quality of service, this PREM helps identify gaps in service delivery and areas for improvement. Healthcare providers can use this information to tailor care to patient needs, fostering more effective communication and personalized support. Additionally, by systematically measuring patient experiences, this tool enables continuous quality improvement, allowing clinics to track progress and implement targeted interventions. Ultimately, integrating this PREM into routine practice has the potential to enhance patient satisfaction, promote better adherence to treatment plans and improve overall health outcomes for individuals with diabetes in Thailand.

## Author Contributions

**Soe Sandi Tint:** conceptualization, methodology, investigation, data curation, formal analysis, writing – original draft. **Nida Buawangpong:** investigation, validation, writing – review and editing. **Wichuda Jiraporncharoen:** investigation, validation, writing – review and editing. **Myo Zin Oo:** validation, writing – review and editing. **Nutchar Wiwatkunupakarn:** validation, writing – review and editing. **Kittipan Rerkasem:** validation, writing – review and editing. **Kanokwan Kulprachakarn:** validation, writing – review and editing. **Hataichanok Chuljerm:** validation, writing – review and editing. **Timothy E. O'Brien:** validation, writing – review and editing. **Rohini Mathur:** validation, writing – review and editing. **Petch Rawdaree:** validation, writing – review and editing. **Chaisiri Angkurawaranon:** conceptualization, supervision, funding acquisition, validation, writing – review and editing.

## Ethics Statement

This study was approved by the Research Ethics Committee of the Faculty of Medicine, Chiang Mai University, Thailand, on 21 February 2024 (No: 072/2024). Before the interview, a written informed consent form was obtained from all patients who wished to continue in the study. Participation in this project was entirely voluntary, and participants were informed that they could withdraw from the study at any point without having to provide a reason.

## Conflicts of Interest

The authors declare no conflicts of interest.

## Supporting information

Supplementary_file_1.

Supplementary_file_2.

Supplementary_file_3.

## Data Availability

The data supporting the findings of this study can be obtained from the corresponding author upon reasonable request and subsequent review by the author.

## References

[1] C. Bull , H. Teede , D. Watson , and E. J. Callander , “Selecting and Implementing Patient‐Reported Outcome and Experience Measures to Assess Health System Performance,” JAMA Health Forum 3, no. 4 (2022): e220326. 36218960 10.1001/jamahealthforum.2022.0326

[2] J. J. Mira , R. Nuño‐Solinís , M. Guilabert‐Mora , et al., “Development and Validation of an Instrument for Assessing Patient Experience of Chronic Illness Care,” International Journal of Integrated Care 16, no. 3 (2016): 13. 10.5334/ijic.2443 PMC5350641 28435422

[3] V. Lowry , V. Tremblay‐Vaillancourt , P. Beaupré , et al., “How Patient‐Reported Outcomes and Experience Measures (PROMs and PREMs) Are Implemented in Healthcare Professional and Patient Organizations? An Environmental Scan,” Journal of Patient‐Reported Outcomes 8, no. 1 (2024): 133. 39546094 10.1186/s41687-024-00795-9 PMC11568099

[4] S. Sofaer , C. Crofton , E. Goldstein , E. Hoy , and J. Crabb , “What Do Consumers Want to Know About the Quality of Care in Hospitals?,” Health Services Research 40 (2005): 2018–2036. 16316436 10.1111/j.1475-6773.2005.00473.x PMC1361244

[5] K. I. Pettersen , “The Patient Experiences Questionnaire: Development, Validity, and Reliability,” International Journal for Quality in Health Care 16, no. 6 (2004): 453–463. 15557355 10.1093/intqhc/mzh074

[6] A. Smith , N. Hex , and M. Taylor , Patient‐Reported Experience Measures (PREMs): A Scoping Document to Inform the Evaluation of the NHS Vanguard Sites (York Health Economics Consortium, 2015).

[7] R. E. Glasgow , E. H. Wagner , J. Schaefer , L. D. Mahoney , R. J. Reid , and S. M. Greene , “Development and Validation of the Patient Assessment of Chronic Illness Care (PACIC),” Medical Care 43, no. 5 (2005): 436–444. 15838407 10.1097/01.mlr.0000160375.47920.8c

[8] Institute for Accreditation of Healthcare Facilities (Public Organization). Patient Experience Program (PEP), 2024 [cited 2025 Feb 14], https://pex.ha.or.th/index.

[9] American Diabetes Association , “Standards of Medical Care in Diabetes—2024,” Diabetes Care 47 (2024): 1–176.

[10] S. S. Tint , M. Z. Oo , N. Buawangpong , et al., “Instruments for Assessing Patient‐Reported Experience Measures Among Patients With Diabetes Mellitus: A Scoping Review,” Journal of Patient‐Reported Outcomes 9, no. 1 (2025): 16. 39921791 10.1186/s41687-025-00848-7 PMC11807032

[11] C. B. Terwee , S. D. M. Bot , M. R. de Boer , et al., “Quality Criteria Were Proposed for Measurement Properties of Health Status Questionnaires,” Journal of Clinical Epidemiology 60, no. 1 (2007): 34–42. 17161752 10.1016/j.jclinepi.2006.03.012

[12] J. Somanawat , K. Saramunee , and S. Chanasopon , “Process, Quality and Challenges of Diabetes Care in Primary Care: A Study of District Health Network in Thailand,” Primary Health Care Research & Development 21 (2020): e46. 33106200 10.1017/S1463423620000468 PMC7681172

[13] World Health Organization. Bureau of Non‐Communicable Diseases. National Strategic Plan for Prevention and Control of Non‐Communicable Diseases, 5 years (2017‐2021), Bangkok, Thailand, 2017 [cited 2025 Feb 14], www.searo.who.int/thailand/areas/national-ncd-prevention-and-control-plan-2017-2021-tha.pdf.

[14] P. Sibounheuang , P. S. Olson , and P. Kittiboonyakun , “Patients’ and Healthcare Providers’ Perspectives on Diabetes Management: A Systematic Review of Qualitative Studies,” Research in Social & Administrative Pharmacy 16, no. 7 (2020): 854–874, https://www.nhso.go.th/en/component/content/article/nhso-annual-report-fiscal-year-2022?catid=36&Itemid=344. 31542445 10.1016/j.sapharm.2019.09.001

[15] B. O. Kirk , R. Khan , D. Davidov , U. Sambamoorthi , and R. Misra , “Exploring Facilitators and Barriers to Patient‐Provider Communication Regarding Diabetes Self‐Management,” PEC Innovation 3 (2023): 100188. 37457669 10.1016/j.pecinn.2023.100188 PMC10339241

[16] National Health Security Office. NHSO Annual Report Fiscal Year 2022. 2023.

[17] G. O. Boateng , T. B. Neilands , E. A. Frongillo , H. R. Melgar‐Quiñonez , and S. L. Young , “Best Practices for Developing and Validating Scales for Health, Social, and Behavioral Research: A Primer,” Frontiers in Public Health 6 (2018): 149. 29942800 10.3389/fpubh.2018.00149 PMC6004510

[18] J. Derriennic , P. Nabbe , M. Barais , et al., “A Systematic Literature Review of Patient Self‐Assessment Instruments Concerning Quality of Primary Care in Multiprofessional Clinics,” Family Practice 39, no. 5 (2022): 951–963. 35230419 10.1093/fampra/cmac007 PMC9508876

[19] M. S. B. Yusoff , “ABC of Content Validation and Content Validity Index Calculation,” Education in Medicine Journal 11, no. 2 (2019): 49–54.

[20] *Multivariate Statistical Methods: A Primer*. 5th ed. Chapman and Hall/CRC; 2024. 10.1201/9781003453482.

[21] M. C. Howard , “A Review of Exploratory Factor Analysis Decisions and Overview of Current Practices: What We Are Doing and How Can We Improve?,” International Journal of Human‐Computer Interaction 32, no. 1 (2016): 51–62.

[22] D. Goretzko , K. Siemund , and P. Sterner , “Evaluating Model Fit of Measurement Models in Confirmatory Factor Analysis,” Educational and Psychological Measurement 84, no. 1 (2024): 123–144. 38250508 10.1177/00131644231163813 PMC10795573

[23] M. Brunner and H.‐M. SÜβ , “Analyzing the Reliability of Multidimensional Measures: An Example From Intelligence Research,” Educational and Psychological Measurement 65, no. 2 (2005): 227–240.

[24] L. B. Mokkink , C. A. C. Prinsen , L. M. Bouter , H. C. W. Vet , and C. B. Terwee , “The Consensus‐Based Standards for the Selection of Health Measurement Instruments (COSMIN) and How to Select an Outcome Measurement Instrument,” Brazilian Journal of Physical Therapy 20, no. 2 (2016): 105–113. 26786084 10.1590/bjpt-rbf.2014.0143 PMC4900032

[25] P. Schober , C. Boer , and L. A. Schwarte , “Correlation Coefficients: Appropriate Use and Interpretation,” Anesthesia & Analgesia 126, no. 5 (2018): 1763–1768. 29481436 10.1213/ANE.0000000000002864

[26] K. Dilbeck , “Factor Analysis: Varimax Rotation,” in The SAGE Encyclopedia of Communication Research Methods, Vol. 4 (SAGE Publications, 2017), 532–533, 10.4135/9781483381411.n191.

[27] J. Sigudla and J. E. Maritz , “Exploratory Factor Analysis of Constructs Used for Investigating Research Uptake for Public Healthcare Practice and Policy in a Resource‐Limited Setting, South Africa,” BMC Health Services Research 23, no. 1 (2023): 1423. 38102600 10.1186/s12913-023-10165-8 PMC10724913

[28] J. F. Hair , G. T. M. Hult , C. M. Ringle , M. Sarstedt , N. P. Danks , and S. Ray , Partial Least Squares Structural Equation Modeling (PLS‐SEM) Using R: A Workbook. (Springer International Publishing, 2021), 1–29.

[29] NHS Digital . National Diabetes Audit – Patient Experience of Diabetes Services Survey 2013‐2014 Pilot Report. 2014 [cited 2025 Feb 20]. https://digital.nhs.uk/data-and-information/publications/statistical/national-diabetes-audit/nda-patient-experience-of-diabetes-services-survey-2013-2014-pilot-report.

[30] M. Svedbo Engström , J. Leksell , U. B. Johansson , et al., “A Disease‐Specific Questionnaire for Measuring Patient‐Reported Outcomes and Experiences in the Swedish National Diabetes Register: Development and Evaluation of Content Validity, Face Validity, and Test‐Retest Reliability,” Patient Education and Counseling 101, no. 1 (2018): 139–146. 28736071 10.1016/j.pec.2017.07.016

[31] M. Claramita , M. D. F. Nugraheni , J. van Dalen , and C. van der Vleuten , “Doctor‐Patient Communication in Southeast Asia: A Different Culture?,” Advances in Health Sciences Education 18, no. 1 (2013): 15–31. 22314942 10.1007/s10459-012-9352-5 PMC3569576

[32] M. Claramita , A. Utarini , H. Soebono , J. Van Dalen , and C. Van der Vleuten , “Doctor‐Patient Communication in a Southeast Asian Setting: The Conflict Between Ideal and Reality,” Advances in Health Sciences Education 16, no. 1 (2011): 69–80. 20658353 10.1007/s10459-010-9242-7 PMC3074074

[33] J. Martin‐Delgado , A. Mula , M. Guilabert , et al., “Development and Validation in Ecuador of the EPD Questionnaire, a Diabetes‐Specific Patient‐Reported Experience and Outcome Measure: A Mixed‐Methods Study,” Health Expectations 25, no. 5 (2022): 2134–2146. 34585477 10.1111/hex.13366 PMC9615093

[34] P. Srichan , T. Apidechkul , R. Tamornpark , et al., “Stigma Experiences and Adaptations in Accessing Healthcare Services Among Hill Tribes in Thailand: A Qualitative Study,” PLoS One 20, no. 5 (May 2025): e0321119. 40315248 10.1371/journal.pone.0321119 PMC12047764

[35] P. S. Olson , C. Ploylearmsang , P. Sibounheuang , et al., “Development of a Patient Satisfaction Questionnaire (PSQ) for Diabetes Management in Thailand and Lao PDR,” PLoS One 19, no. 3 (2024): e0300052. 38452151 10.1371/journal.pone.0300052 PMC10919862

[36] Agency for Healthcare Research and Quality. What is patient experience? [cited 2025 Feb 20]. https://www.ahrq.gov/cahps/about-cahps/patient-experience/index.html.

[37] S. Steine , A. Finset , and E. Laerum , “A New, Brief Questionnaire (PEQ) Developed in Primary Health Care for Measuring Patients’ Experience of Interaction, Emotion, and Consultation Outcome,” Family Practice 18, no. 4 (2001): 410–418. 11477049 10.1093/fampra/18.4.410

[38] American Diabetes Association ,Introduction to Your Rights and Care Standards: A Guide for People With Type 2 Diabetes,” Clinical Diabetes: A Publication of the American Diabetes Association 43, no. 3 (2025): 334. 40741464 10.2337/cd25-pint PMC12304558

[39] J. Burt , F. S. Mair , and C. R. May , “Expanded Care Planning Paradigm for Older Adults With Type 2 Diabetes,” Journal of Diabetes and its Complications 39, no. 6 (2025): 1077–1083.

[40] M. Tavakol and R. Dennick , “Making Sense of Cronbach's Alpha,” International Journal of Medical Education 2 (June 2011): 53–55. 28029643 10.5116/ijme.4dfb.8dfd PMC4205511

