# Supplementary material for: Development and Validation of a Culturally Adapted Patient‐Reported Experience Measure for Diabetes Care in Thailand: Mixed‐Methods Study
Source: Health Expect. 2026 Feb 27;29(2):e70619. doi: 10.1111/hex.70619 (PMC12947248; doi:10.1111/hex.70619)
Supplement: Supplementary file 1 — Supplementary_file_1. [file HEX-29-e70619-s001.docx]

**Appendix 1: Exploratory Factor Analysis Iterations: Item Reduction Process Before Final 18-Item Solution**

**Step 1: Exploratory Factor Analysis Results for the Initial 22 PREM Items**

| **No** | **Variable** | **Factor1** | **Factor2** | **Factor3** | **Factor4** | **Factor5** | **Factor6** | **Decision** |
| --- | --- | --- | --- | --- | --- | --- | --- | --- |
| 1. | Did you agree on your care planning with your health care provider about the goal setting of your diabetes care? **goal_q2** | 0.2895 | -0.0658 | **0.5355** | -0.1976 | 0.2951 | 0.0817 |  |
| 2. | Were you offered a written, printed, or electronic copy of your care plan? **goal_q3** | -0.0603 | -0.0586 | 0.0448 | -0.1142 | **0.8371** | 0.0307 |  |
| 3. | Did you agree on your care planning with your health care provider about a plan for lifestyle modification? **life_q3** | -0.0514 | 0.1369 | **0.906** | 0.1456 | -0.0703 | -0.0219 |  |
| 4. | Did you receive useful and enough information from the health care provider about your disease condition? **dis_condit2** | 0.3529 | -0.1096 | 0.3892 | 0.011 | **0.4124** | 0.3917 |  |
| 5. | To what extent did you receive useful and sufficient information on how to handle if you have symptoms of too high or too low blood glucose level? **mx_q2** | 0.0298 | **0.4912** | 0.2938 | 0.279 | 0.2693 | 0.3205 |  |
| 6. | Did you receive useful and enough useful information from the health care provider about the lifestyle modification? **life_q2** | 0.1248 | 0.1432 | **0.8944** | 0.0199 | 0.05 | 0.0467 |  |
| 7. | Did you receive useful and enough information from the health care provider about the psychological impact of diabetes on your daily life? **psy_q2** | 0.0853 | 0.31 | 0.0964 | 0.0354 | 0.038 | **0.7889** |  |
| 8. | Did you receive useful and enough information from health care provider about the diabetes medication and treatment? **tx_q2** | **0.4341** | 0.0272 | **0.6187** | -0.0791 | 0.2949 | 0.1997 |  |
| 9. | To what extent did you receive useful information about the medical devices that you used at your home? (eg. glucometer, insulin pen) **device_q2** | 0.2771 | 0.1329 | -0.2851 | **0.4057** | 0.0253 | -0.0918 | **Removed** |
| 10. | To what extent did your healthcare provider explain diabetes-related information to you clearly? **pro_q1** | **0.4933** | **0.5581** | 0.2309 | 0.1079 | -0.1663 | -0.1521 |  |
| 11. | To what extent did your healthcare provider listen carefully to your concerns and questions? **pro_q2** | 0.0201 | **0.9385** | 0.0557 | 0.0121 | -0.0451 | 0.1281 |  |
| 12. | To what extent did your healthcare provider effectively address your concerns and questions about diabetes management? **pro_q3** | 0.0623 | **0.9328** | 0.0815 | 0.1048 | -0.0154 | 0.0669 |  |
| 13. | To what extent did you feel respected and treated with dignity during your interactions with healthcare providers? **pro_q4** | 0.2378 | **0.5167** | 0.1815 | 0.2823 | **0.4663** | -0.1544 |  |
| 14. | To what extent did you have confidence and trust in your healthcare provider managing your diabetes? **pro_q5** | 0.3716 | 0.3805 | **0.4031** | 0.3235 | 0.1988 | **-0.4182** | **Removed** |
| 15. | To what extent did your healthcare provider coordinate well with other specialists involved in your diabetes care? **pro_q6** | 0.1821 | 0.1522 | 0.1269 | **0.8656** | -0.0599 | -0.0345 |  |
| 16. | How satisfied are you with the accessibility of the care structure? (e.g., transportation, parking, etc.) **qos_q1** | **0.7754** | -0.0749 | 0.1617 | 0.1811 | 0.0404 | 0.087 |  |
| 17. | How satisfied are you with the clinic environment (e.g., cleanliness, comfort, and overall atmosphere) **qos_q2** | **0.8273** | 0.0899 | 0.0529 | -0.0163 | -0.1228 | -0.0463 |  |
| 18. | How satisfied are you with the communication with the healthcare provider during the consultation?**qos_q3** | **0.7237** | 0.3013 | 0.1086 | 0.2834 | -0.0715 | -0.1557 |  |
| 19. | How satisfied are you with the overall waiting experience, including wait times before seeing the doctor, during consultation, and after seeing the doctor (to get medicine, to pay at the cashier)? **qos_q4** | **0.5722** | 0.0104 | -0.1383 | **0.4266** | 0.3938 | 0.0132 |  |
| 20. | How satisfied are you with the communication with the other clinic staff during your visit? **qos_q5** | **0.6325** | 0.3941 | 0.0605 | 0.0744 | 0.1875 | 0.0411 |  |
| 21. | How satisfied are you with the accessibility of your health care provider when you need help with diabetes care? (e.g., contact number, appointment when needed)**qos_q6** | **0.5421** | 0.0777 | 0.0167 | **0.5824** | -0.1026 | 0.2753 |  |
| 22. | How satisfied are you with the range of diabetes care services provided, such as blood tests, eye exams, and foot checks? **qos_q7** | **0.7819** | 0.0079 | 0.1341 | 0.1947 | 0.2033 | 0.1809 |  |

**Step 2: Exploratory Factor Analysis Results for the 20 PREM Items**

| **No.** | **Variable** | **Factor1** | **Factor2** | **Factor3** | **Factor4** | **Decision** |
| --- | --- | --- | --- | --- | --- | --- |
| 1. | Did you agree on your care planning with your health care provider about the goal setting of your diabetes care? **goal_q2** | 0.0341 | **0.5476** | -0.2246 | 0.1809 |  |
| 2. | Were you offered a written, printed, or electronic copy of your care plan? **goal_q3** | -0.0185 | -0.1835 | -0.0556 | **0.7271** |  |
| 3. | Did you agree on your care planning with your health care provider about a plan for lifestyle modification? **life_q3** | 0.1669 | **0.8042** | 0.2374 | -0.0567 |  |
| 4. | Did you receive useful and enough information from the health care provider about your disease condition?**dis_condit2** | 0.1683 | **0.4862** | -0.0923 | **0.5056** |  |
| 5. | To what extent did you receive useful and sufficient information on how to handle if you have symptoms of too high or too low blood glucose level? **mx_q2** | 0.1966 | 0.3123 | 0.3596 | 0.3867 | **Removed** |
| 6. | Did you receive useful and enough useful information from the health care provider about the lifestyle modification? **life_q2** | 0.1161 | **0.8179** | 0.2688 | -0.0623 |  |
| 7. | Did you receive useful and enough information from the health care provider about the psychological impact of diabetes on your daily life? **psy_q2** | 0.0098 | 0.098 | 0.35 | **0.6043** |  |
| 8. | Did you receive useful and enough information from health care provider about the diabetes medication and treatment? **tx_q2** | **0.435** | **0.5649** | -0.0471 | 0.1436 |  |
| 9. | To what extent did your healthcare provider explain diabetes-related information to you clearly? **pro_q1** | **0.4474** | 0.2177 | **0.4067** | -0.045 |  |
| 10. | To what extent did your healthcare provider listen carefully to your concerns and questions? **pro_q2** | 0.1389 | 0.1275 | **0.8648** | 0.0103 |  |
| 11. | To what extent did your healthcare provider effectively address your concerns and questions about diabetes management? **pro_q3** | 0.1741 | 0.1286 | **0.8332** | 0.0429 |  |
| 12. | To what extent did you feel respected and treated with dignity during your interactions with healthcare providers? **pro_q4** | **0.5081** | 0.1756 | **0.4808** | 0.1259 |  |
| 13. | To what extent did your healthcare provider coordinate well with other specialists involved in your diabetes care? **pro_q6** | **0.4999** | 0.1263 | 0.3422 | -0.0665 | **Removed** |
| 14. | How satisfied are you with the accessibility of the care structure? (e.g., transportation, parking, etc.) **qos_q1** | **0.7335** | 0.1613 | 0.0015 | 0.0336 |  |
| 15. | How satisfied are you with the clinic environment (e.g., cleanliness, comfort, and overall atmosphere) **qos_q2** | **0.7086** | 0.0742 | 0.1902 | -0.0343 |  |
| 16. | How satisfied are you with the communication with the healthcare provider during the consultation?**qos_q3** | **0.745** | 0.159 | 0.3071 | -0.1011 |  |
| 17. | How satisfied are you with the overall waiting experience, including wait times before seeing the doctor, during consultation, and after seeing the doctor (to get medicine, to pay at the cashier)? **qos_q4** | **0.7168** | -0.0633 | -0.0129 | 0.2329 |  |
| 18. | How satisfied are you with the communication with the other clinic staff during your visit? **qos_q5** | **0.7417** | 0.1599 | 0.2812 | 0.0595 |  |
| 19. | How satisfied are you with the accessibility of your health care provider when you need help with diabetes care? (e.g., contact number, appointment when needed)**qos_q6** | **0.5879** | 0.3083 | 0.1825 | 0.0004 |  |
| 20. | How satisfied are you with the range of diabetes care services provided, such as blood tests, eye exams, and foot checks? **qos_q7** | **0.8022** | 0.1729 | 0.0794 | 0.0181 |  |
