# Supplementary material for: Development and Validation of a Culturally Adapted Patient‐Reported Experience Measure for Diabetes Care in Thailand: Mixed‐Methods Study
Source: Health Expect. 2026 Feb 27;29(2):e70619. doi: 10.1111/hex.70619 (PMC12947248; doi:10.1111/hex.70619)
Supplement: Supplementary file 3 — Supplementary_file_3. [file HEX-29-e70619-s003.pdf]

Section 1: Patient Information

This section collects basic information about you, such as your age, gender, and medical history. Please put a √ in the ☐ and write the answers in the spaces provided below .

No	Question	Answer
1.1	Age ________________________year	
1.2	Gender<br>☐ 1. Male<br>☐ 2. Female	
1.3	Marital status<br>☐ 1. Single Never-Married<br>☐ 2. Married<br>☐ 3. Widowed/<br>4. Divorced	
1.4	Education status<br>☐ 1. No formal education<br>☐ 2. Primary education (Grade 1 to 6)<br>☐ 3. Lower secondary<br>☐ 4. Secondary<br>5. Bachelor<br>6. Master	
1.5	What is your current work?<br>☐ 1. Government staff<br>☐ 2. Private company staff<br>☐ 3. Own business<br>☐ 4. Migrant worker<br>☐ 5. Other (please specify) _________________	
1.6	Period of diagnosis of diabetes ________ years _______ months.	
1.7	Other chronic conditions	

Section 2: Patient Reported Experiences on Diabetes Care

This section evaluates your experience with diabetes care services during your last appointment, organized into four parts:

- **Parts A and B:** These parts address the domains of **Care Planning and Patient Education.** They include combined questions for their shared sub-domains.
- **Part C:** This part focuses on the domain of **Professionalism** and includes specific items related to this area.
- **Part D:** This part covers **Quality of Services** and includes items related to this domain.

Please read the statements in each part carefully. Your feedback is valuable and helps us improve our services. Answer each question honestly using the provided scale (e.g., 1 to 5), and mark your answers with a '√'. Your responses are confidential and will only be used to enhance our services. Thank you!

Part A: Care Planning and Part B: Patient Education Part C: Professionalism

This part is a questionnaire that assesses your experience with care planning and patient education during your consultation. The scoring criteria are as follows:

Score 1: Not at all: The statement does not apply to my experience in any way.

Score 2: To a small extent: The statement applies to my experience but only minimally.

Score 3: To a moderate extent: The statement somewhat applies to my experience.

Score 4: To a large extent: The statement mostly applies to my experience.

		Not at all (1)	To a small extent (2)	To moderate extent (3)	To a large extent (4)	To a very large extent (5)
**1.**	**Care Planning**					
1.1	To what extent did you **agree on your blood glucose level targets** that you and your healthcare provider set together?"					
1.2	To what extent did you **agree** with your **lifestyle modification plan** that you and your healthcare provider set together?"					
**2.**	**Patient Education**					
2.1	To what extent did you **receive** useful and sufficient **information about your diabetes** from your healthcare provider such as its causes, symptoms, and progression?					
2.2	To what extent did you **receive** useful **information about lifestyle modifications** such as diet, physical activity, foot care, and medication adherence from your healthcare provider?					
2.3	To what extent did you **receive useful information about** the **psychological impact of diabetes** on your daily routine life? (eg. angry, problems with sleeping, easy to cry, stress, etc.)					
2.4	To what extent did you **receive** useful **information about your current medication,** including any changes in doses or types?					
**3.**	**Professionalism**					
3.1	To what extent did your healthcare provider explain diabetes-related information to you clearly?					
3.2	To what extent did your healthcare provider listen carefully to your concerns and questions?					
3.3	To what extent did you feel respected and treated with dignity during your interactions with healthcare providers?					

Part D: Quality of service

This part is a questionnaire that assesses your experience with the quality of service during your consultation. The scoring criteria are as follows:

Score 1: Very Dissatisfied: The service or experience was extremely unsatisfactory.

Score 2: Dissatisfied: The service or experience was somewhat unsatisfactory.

Score 3: Neutral: The service or experience was neither satisfactory nor unsatisfactory.

Score 4: Satisfied: The service or experience was generally satisfactory.

Score 5: Very Satisfied: The service or experience was extremely satisfactory.

		Very dissatisfied (1)	Dissatisfied (2)	Neutral (3)	Satisfied (4)	Very satisfied (5)
1.	How satisfied are you with the accessibility of the care structure? (eg. transportation, parking, etc..)					
2.	How satisfied are you with the clinic environment (eg. cleanliness, comfort, and overall atmosphere)					
3.	How satisfied are you with the communication with the healthcare provider during the consultation?					
4.	How satisfied are you with the overall waiting experience, including wait times before seeing the doctor, during consultation, and after seeing the doctor (to get medicine, to pay at the cashier)?					
5.	How satisfied are you with the communication with the other clinic staff during your visit?					
6.	How satisfied are you with the accessibility of your health care provider when you need help with diabetes care? (eg. contact number, appointment when needed)					
7.	How satisfied are you with the range of diabetes care services provided, such as blood tests, eye exams, and foot checks?					

ประสบการณ์ในการเข้ารับบริการคลินิกเบาหวานตามนัดครั้งที่ผ่านมา

คำชี้แจง: ส่วนนี้จะประเมินประสบการณ์ของท่านระหว่างการให้คำพูดคุยปรึกษาในการเข้ารับบริการคลินิกเบาหวานตามนัดครั้งที่ผ่านมา แบ่งออกเป็น 4 ด้าน ได้แก่ ก) ด้านการวางแผนการดูแลรักษา ข) ด้านการให้ความรู้แก่ผู้ป่วย ค) ด้านความเป็นมืออาชีพ ง) ด้านคุณภาพการให้บริการ

โปรดพิจารณาคำถามในแต่ละข้อและให้ระดับคะแนนที่ให้ไว้ (เช่น 1 ถึง 5) และทำเครื่องหมาย "√"

ด้าน ก ข และ ค : การวางแผนการดูแลรักษา การให้ความรู้แก่ผู้ป่วย และความเป็นมืออาชีพ

เกณฑ์การให้คะแนนมีดังนี้:

1 คะแนน : ไม่มี หมายถึง ข้าพเจ้าไม่ได้รับประสบการณ์เลย

2 คะแนน : เล็กน้อย หมายถึง ข้าพเจ้าได้รับประสบการณ์เพียงเล็กน้อยเท่านั้น

3 คะแนน : ปานกลาง หมายถึง ข้าพเจ้าได้รับประสบการณ์ปานกลาง

4 คะแนน : มาก หมายถึง ข้าพเจ้าได้รับประสบการณ์มาก

5 คะแนน : มากที่สุด หมายถึง ข้าพเจ้าได้รับประสบการณ์มากที่สุด

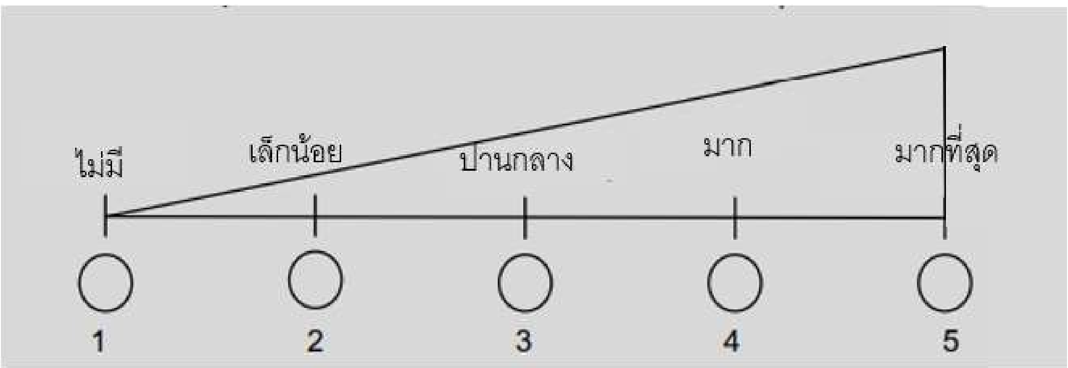

ด้าน ก ข และ ค		ระดับคะแนน				
		ไม่มี	เล็กน้อย	ปานกลาง	มาก	มากที่สุด
**1.การวางแผนการดูแลรักษา**						
1.1	ท่าน**เห็นด้วย**กับ**เป้าหมายระดับน้ำตาลในเลือด**ที่ตั้งไว้ร่วมกับบุคลากรทางการแพทย์มากน้อยเพียงใด?					
1.2	ท่าน**เห็นด้วย**กับ**แผนการปรับเปลี่ยนพฤติกรรมสุขภาพ** ที่ตั้งร่วมกับบุคลากรทางการแพทย์ มากน้อยเพียงใด?					
**2. การให้ความรู้แก่ผู้ป่วย**						
2.1	ท่าน**ได้รับข้อมูล**ที่เป็นประโยชน์เพียงพอเกี่ยวกับ**โรคเบาหวาน**ของท่านจากบุคลากรทางการแพทย์มากน้อยเพียงใด เช่น สาเหตุของโรค, อาการ, และการดำเนินของโรค					
2.2	ท่าน**ได้รับข้อมูล**ที่เป็นประโยชน์จากบุคลากรทางการแพทย์เกี่ยวกับ**การปรับเปลี่ยนพฤติกรรมสุขภาพ** มากน้อยเพียงใด เช่น การกินอาหาร การมีกิจกรรมทางกาย การดูแลเท้า การกินยา การดื่มสุรา การสูบบุหรี่ หรืออื่น ๆ มากน้อยเพียงใด?					
2.3	ท่าน**ได้รับข้อมูล**ที่เป็นประโยชน์เกี่ยวกับ**ผลกระทบทางจิตใจจากโรคเบาหวานหรือวิธีการจัดการ** มากน้อยเพียงใด เช่น โกรธ มีปัญหาเกี่ยวกับการนอนหลับ เครียด					
2.4	ท่าน**ได้รับข้อมูล**ที่เป็นประโยชน์เกี่ยวกับ**การใช้ยารักษาโรคเบาหวาน** มากน้อยเพียงใด?					
**3.ความเป็นมืออาชีพ**						
3.1	บุคลากรทางการแพทย์สามารถ**อธิบายข้อมูล**เกี่ยวกับโรคเบาหวานให้ท่านเข้าใจได้ง่าย มากน้อยเพียงใด?					
3.2	บุคลากรทางการแพทย์**รับฟังข้อกังวลหรือคำถามที่ท่านสงสัยอย่างตั้งใจ** มากน้อยเพียงใด?					
3.3	ท่าน**ได้รับความเคารพและการปฏิบัติอย่างมีศักดิ์ศรี** ในระหว่างพบบุคลากรทางการแพทย์มากน้อยเพียงใด?					

ด้าน ง : ด้านคุณภาพการให้บริการ

แบบสอบถามส่วนนี้เป็นการประเมินเกี่ยวกับคุณภาพการให้บริการระหว่างที่ท่านพูดคุยปรึกษากับบุคลากรทางการแพทย์

เกณฑ์การให้คะแนนมีดังนี้:

1 คะแนน : ไม่พอใจมาก หมายถึง การบริการหรือประสบการณ์ไม่น่าพอใจอย่างยิ่ง

2 คะแนน : ไม่พอใจ หมายถึง การบริการหรือประสบการณ์ค่อนข้างไม่น่าพอใจ

3 คะแนน : ปานกลาง หมายถึง การบริการหรือประสบการณ์ไม่เป็นที่น่าพอใจหรือไม่น่าพอใจ

4 คะแนน : พอใจ หมายถึง การบริการหรือประสบการณ์โดยทั่วไปเป็นที่น่าพอใจ

5 คะแนน : พอใจมาก หมายถึง การบริการหรือประสบการณ์เป็นที่น่าพอใจอย่างยิ่ง

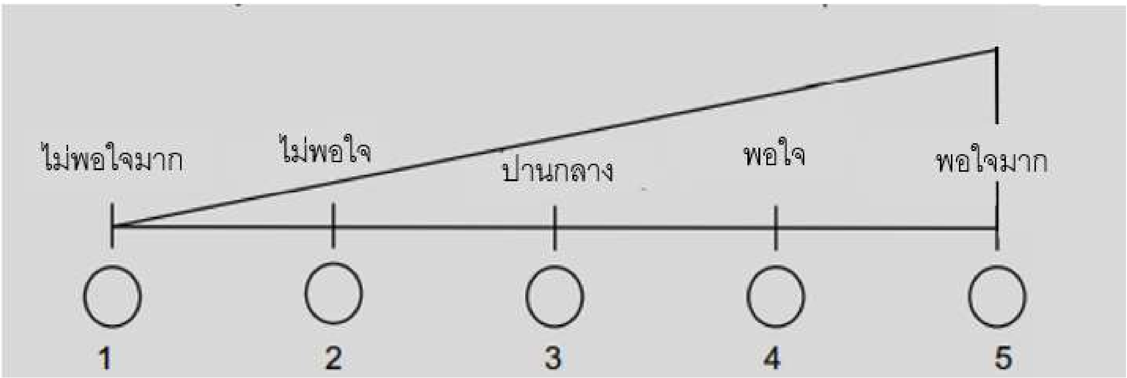

**ด้าน ง : ด้านคุณภาพการให้บริการ**		ระดับคะแนน				
		ไม่พอใจมาก	ไม่พอใจ	ปานกลาง	พอใจ	พอใจมาก
1.	**ความสะดวก**ของการเข้ารับการรักษา (เช่น การเดินทาง ที่จอดรถ ฯลฯ..)					
2.	**สภาพแวดล้อม**โดยรวมของคลินิก (เช่น ความสะอาด ความสะดวกสบาย ความปลอดภัย และบรรยากาศโดยรวม)					
3.	**การพูดคุยสื่อสาร**กับ**เจ้าหน้าที่**คลินิกในตอนที่ท่านมาคลินิก (เช่น การทำนัด การลงทะเบียน)					
4.	**ระยะเวลา**ในการมารับบริการเกี่ยวโรคเบาหวาน เช่น เวลารอก่อนพบบุคลากรทางการแพทย์ ระหว่างพูดคุยปรึกษา และหลังพบบุคลากรทางการแพทย์ (ไปรับยา จ่ายเงินที่แคชเชียร์)					

		ระดับคะแนน				
		ไม่พอใจมาก	ไม่พอใจ	ปานกลาง	พอใจ	พอใจมาก
5.	**การพูดคุยสื่อสาร**กับ**บุคลากรทางการแพทย์**ในระหว่างที่ท่านรับการรักษา					
6.	**การเข้าถึง**บุคลากรทางการแพทย์เมื่อท่านต้องการความช่วยเหลือเกี่ยวกับโรคเบาหวาน (เช่น เบอร์ติดต่อ นัดหมายเมื่อจำเป็น)					
7.	**ความครอบคลุม**ของการดูแลโรคเบาหวาน (เช่น การตรวจเลือด การตรวจตา และการตรวจเท้า)					
